# The effect of positioning cations on acidity and stability of the framework structure of Y zeolite

**DOI:** 10.1038/srep23382

**Published:** 2016-03-18

**Authors:** Changshun Deng, Junji Zhang, Lihui Dong, Meina Huang, Bin Li, Guangzhou Jin, Junbin Gao, Feiyue Zhang, Minguang Fan, Luoming Zhang, Yanjun Gong

**Affiliations:** 1 Guangxi Key Laboratory Petrochemical Rescource Processing and Process Intensification Technology, School of Chemistry and Chemical Engineering, Guangxi University, Nanning 530004, P.R. China; 2 Department of Chemical Engineering, Beijing Institute of Petro-Chemical Technology, Beijing 102617, P.R. China; 3 State Key Laboratory of Heavy Oil Processing, The Key Laboratory of Catalysis of CNPC, China University of Petroleum-Beijing, Beijing 102249, P.R. China

## Abstract

The investigation on the modification of NaY zeolite on LaHY and AEHY (AE refers Ca and Sr and the molar ratio of Ca and Sr is 1:1) zeolites was proformed by XRD, N_2_-physisorption (BET), XRF, XPS, NH_3_-TPD, Py-IR, hydrothermal stability, and catalytic cracking test. These results indicate that HY zeolite with ultra low content Na can be obtained from NaY zeolite through four exchange four calcination method. The positioning capability of La^3+^ in sodalite cage is much better than that of AE^2+^ and about 12 La^3+^ can be well coordinated in sodalite cages of one unit cell of Y zeolite. Appropriate acid amount and strength favor the formation of propylene and La^3+^ is more suitable for the catalytic cracking of cyclohexane than that of AE^2+^. Our results not only elaborate the variation of the strong and weak acid sites as well as the Brönsted and Lewis acid sites with the change of exchanged ion content but also explore the influence of hydrothermal aging of LaHY and AEHY zeolites and find the optimum ion exchange content for the most reserved acid sites. At last, the coordination state and stabilization of ion exchanged Y zeolites were discussed in detail.

Y zeolites, one type of the best-defined supported catalysts, consist of metal-atom complexes bonded to supports. Such catalysts have been found to have extensive industrial applications for processes including Fluid Catalytic Cracking (FCC)^1^, glycerol acetylation^2^, hydro-liquefaction^3^, and glucose conversion^4^ and are drawing increased attention because they offer opportunities for new properties combined with the most efficient use of the metals^5^. Among them, NaY zeolite is basic and typical catalyst, but its catalytic ability is poor. Through ion exchange, that is, Na^+^ from the framework are substituted by H^+^ and forms HY zeolite which has very strong acidic center and is excellent catalyst in the acid-catalyzed reaction. However, the hydrothermal stability of HY zeolite is very poor. Thus, they are usually modified through metal ion by many researchers^1^,^3^,^4^,^6^, and good catalytic activity and a certain hydrothermal stability are achieved in response to different catalytic reactions. In addition, Some recent works also reveal that introducing metal ions^7^,^8^ and especially rare earth ions^9^ can improve the photo catalytic activity in the point view of energy level engineering.

In recent decades, with regard to ion exchange, most studies use rare earth metals or basic metals (especially the transition metals) to modify HY or NaY zeolite^4^,^5^,^6^,^10^,^11^. Although catalytic activity and hydrothermal stability are optimized, there exists two questions which involve blind and waste. On the one hand, on the amount of ion exchange, only mass percentage is regarded as measurement method. Gao *et al*.^12^ made a study of the rare earth exchange performance on heavy oil conversion with different content of RE cations, but the correlation of the content of RE cations with acidity was still unknown. Similar phenomena can be found in many literatures^4^,^13^,^14^. This not only can not determine the extent of the rest of H^+^ which are not replaced by metal ions but also makes metal ions gather in supercage and block channels if exchanged ions are excessive. On the other hand, because exchanged extent is not determined, it is easy to lead to the excess of exchanged ions, which not only has negative effect on catalytic activity but also brings about raw materials waste. Besides, a disordered unit cell might also be formed^15^, whose acidity and distribution are more difficult to be determined. In contrast, if the amount of acid can be specifically changed with a purpose according to different reactions, this has a guiding sense to the type of different reactions including acid catalysis, weak acid catalysis and even base catalysis. For example, in the fluid catalytic cracking industry, the fresh catalyst consumption per day may be up to several tons. Lowering the rare earth content of the catalyst is a good way of cutting down the consumption of rare earth elements so as to decrease the final cost of the product and to protect limited rare earth resources^12^. Although the study of modified Y zeolite has been extensively reported, a detailed investigation of modified Y zeolite on changing specific amount of acid determined by the unit cell level as well as the distribution of Lewis and Brönsted acid sites is not found in the literatures. In addition, a detailed experimental survey of the hydrothermal stability of modified Y zeolite has rarely been performed.

In the present work, NaY zeolite was modified into HY zeolite and HY zeolite was exchanged by different amounts of La and Ca and Sr (the molar ratio of Ca and Sr is 1:1) ions, respectively, which was determined by the unit cell of HY zeolite, that is, one La^3+^ or Ca^2+^ and Sr^2+^ substitute an equal amount of H^+^ in a unit cell of HY zeolite. And then, the obtained catalysts were characterized by means of XRD, N_2_-physisorption (BET), XRF, XPS, NH_3_-TPD, Py-IR and hydrothermal stability. And the catalytic properties of partial samples were performed in catalytic cracking of cyclohexane. This study is mainly focused on: (1) exploring the influence and understanding the interaction between the exchanged metal ions and HY zeolite; (2) investigating the change of the amount of acid and the distribution of Lewis and Brönsted acid sites when different amount of H^+^ were exchanged by metal ions in HY zeolite; (3) inspecting the change of the hydrothermal stability of exchanged HY zeolite, which comes into being by different amounts of metal ions.

## Results and Discussion

### Impact on the framework

In order to understand the properties of ion exchanged Y zeolites, it is necessary to know the positions of the metal cations in the zeolite framework^16^,^17^. The Rietveld method of structure refinement allows determining the crystallographic sites occupied by rare earth cations.

**Figure 1:**
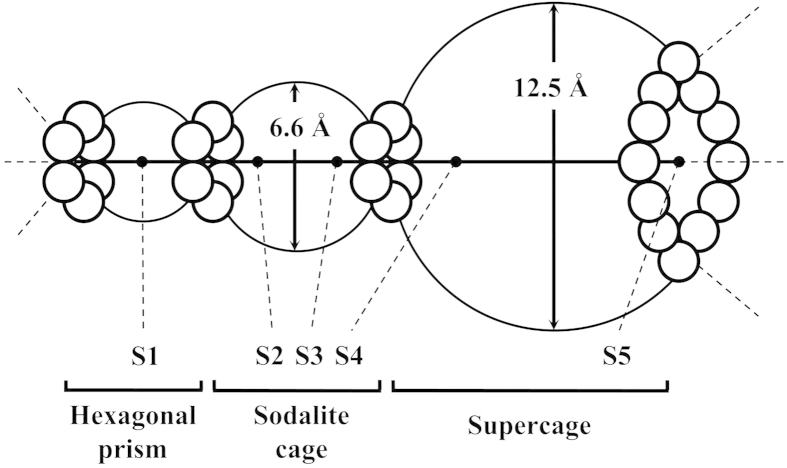
The crystallographic sites for non-framework atoms.

Falabella *et al*.^18^ studied the effect of calcination on the location of La^3+^ and Ce^3+^ in zeolites which have undergone thermal and hydrothermal treatments. They observed that, initially, sodium ions were in sites S2 (centre of the hexagonal prism between the sodalite cage and the supercage^19^) (Fig. 1). This site is usually the most stable and, as consequence, the most populated one since it minimises the electrostatic repulsion between the cations. On the other hand, La^3+^ occupied, especially, S5 (centre of the 12-ring window^19^). According to Frising and Leflaive^19^ this site (S5) seems to be only occupied in rare earth exchanged samples (La^3+^ and Ce^3+^). However, La^3+^ can be frequently found in sites I and I′. Sometimes, after calcinations, rare earth moved to S2, migrating to inside the sodalite cage. The incorporation of RE may induce some structure dealumination, thereby removing aluminium from the framework. These aluminium ions, after treatments, were located in sites S1 (in the hexagonal prism) and the water molecules at sites S3 (in the supercage, close to a square window^19^). Such behaviour is observed regardless of the type of RE atom (La or Ce). Table 1 shows the change of the atoms position before and after calcination.

### Texture and structure characterization (N_2_-physisorption and XRD)

**Figure 2:**
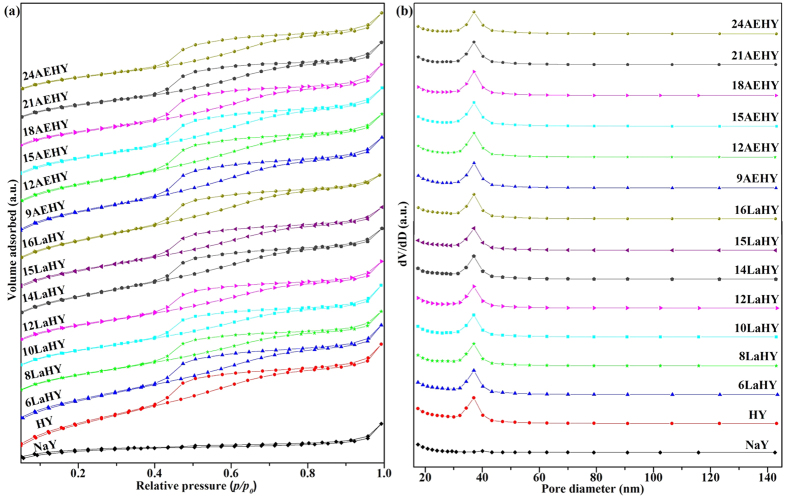
The (**a**) N_2_ adsorption-desorption isotherms, and (**b**) BJH pore size distribution curves of these samples.

Nitrogen adsorption-desorption by a series of ion exchanged HY zeolits was examined in order to estimate their specific surface area and porosity. The results of N_2_ adsorption-desorption analysis are summarized in Fig. 2 for adsorption-desorption isotherms and BJH pore size distribution curves and in Table 2 for surface area and pore volume. Noting from Fig. 2a that all of the samples exhibit the IV-type isotherms with evident H2-type hysteresis loops when NaY zeolite was modified according to IUPAC^20^. This result indicates that HY, LaHY and AEHY zeolites have mesoporous structures, and the H2 hysteresis is typical for a disordered and complex pore structure. It represents pores with narrow and wide sections which are possibly interconnected to each other by channels^20^,^21^. The pore size distribution presented in Fig. 2b shows that these samples possess a unimodal nanoporous structure with narrow pore size range between 32 to 43 nm except NaY zeolite. It can be seen from Table 2 that both the BET surface area and pore volume decrease with the increase of exchanged ion content, indicating the exchanged ions may occupy the porous sites on the HY zeolite and result in lower surface area and smaller pore volume. But both the BET surface area and pore volume of LaHY zeolites decrease slower than that of AEHY zeolites, with the increase of exchanged ion content of La^3+^ and AE^2+^, respectively. Moreover, the pore sizes are almost unchanged when the exchanged content of La^3+^ ranges between HY and 12LaHY on one unit cell of zeolite, the pore sizes increase when the exchanged content of La^3+^ more than 12LaHY. The pore sizes, however, first, show an increasing trend, then decrease gradually with the increase of exchanged ion content of AE^2+^. It has been mentioned previously that metal ions will move to the sodalite cage after calination compared with that before calination whose metal ions almost exist in supercage. Thus, these results indicate that La^3+^ is easier and better moved to and located in sodalite cage compared with AE^2+^. The capability of AE^2+^ moving to sodalite cage is worse and more AE^2+^ will assemble in supercage, which results in stronger disorder of the channels of zeolite and consequensely leads to the further decrease of the BET surface area and pore volume and the complexity of the pore size for AEHY zeolites with the increase of exchanged ion content of AE^2+^.

**Figure 3:**
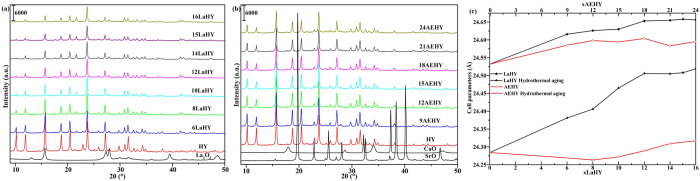
The XRD patterns of (**a**) LaHY, (**b**) AEHY samples and their (**c**) cell parameters of fresh and hydrothermal aging.

In order to study the structure of modified HY zeolites, X-ray diffraction technique had been used to study the crystal structures of series of catalysts. The XRD results are shown in Fig. 3a,b for LaHY and AEHY samples, respectively, and in 3c for their cell parameters of fresh and hydrothermal aging. It can be seen from Fig. 3a,b that all samples exhibit the typical diffraction peaks of the faujasite (FAU) structure^22^,^23^,^24^. Peaks ascribed to La_2_O_3_ for LaHY zeolites and ascribed to CaO and SrO for AEHY zeolites are not observed for corresponding samples, suggesting that La species or Ca and Sr species have entered the structures of the Y zeolites^25^,^26^. The peak intensities of both LaHY and AEHY zeolites gradually decrease with the increase of the exchanged ion content of La^3+^ and AE^2+^ compared with HY zeolite, which results from that La species or Ca and Sr species have entered into the channels of Y zenlites. But it should be noted that the reducing amplitude of the peak intensities of LaHY zeolite is larger than that of AEHY zeolite when the same amount of H^+^ are exchanged by metal ions in HY zeolite. In fact, for Y zeolite, periodic arrangement of skeletal atoms makes X-ray form diffraction, the difference is that there exits cations which are used for the balance of skeleton negative charge in the channels of zeolite compared with crystal. The arrangement of extraframework cations are more disordered with respect to skeleton atoms in zeolite, and the electrons of these cations can also arise vibration and form X-ray when they are radiated by X-ray, which reduces the accurateness of zeolite parameters. The influence is minor when the balance cations are Na^+^, but the effect is great when the balance cations are rare earth cations. Thus, the above phenomenon appears. It can be seen from Fig. 3c that for fresh samples, the cell parameters of both LaHY and AEHY zeolites gradually increase until 12LaHY or 18AEHY zeolite with the increase of the exchanged ion content of La^3+^ or AE^2+^, but further increase the exchanged ion content the cell parameters only increase slightly. Especially noteworthy is that the increasing amplitude of the cell parameters of LaHY zeolite is larger than that of AEHY zeolite when the same amount of H^+^ are exchanged by metal ions in HY zeolite. On the other hand, the ionic radii of the three ions are almost the same (La^3+^: 1.02 Å, Ca^2+^: 0.99 Å, Sr^2+^: 1.12 Å). Both of which indicate that La^3+^ is easier moved to sodalite cage and coordinates with framework O3^26^ (which leads to cell expansion) than that of AE^2+^ after calination. Furthermore, when the ion exchange content exceeds 12 La^3+^ or 18 E^2+^ in one unit cell of Y zeolite, the cell parameters only increase slightly, indicating that the ion content in sodalite cage can almost reach saturation when the ion exchange content reach 12 La^3+^ or 18 AE^2+^ in one unit cell of Y zeolite, similar results have been reported by Li *et al*.^27^. In short, the XRD results are in good agreement with the N_2_-physisorption results.

### Element analysis and surface state characterization (XRF and XPS)

**Figure 4:**
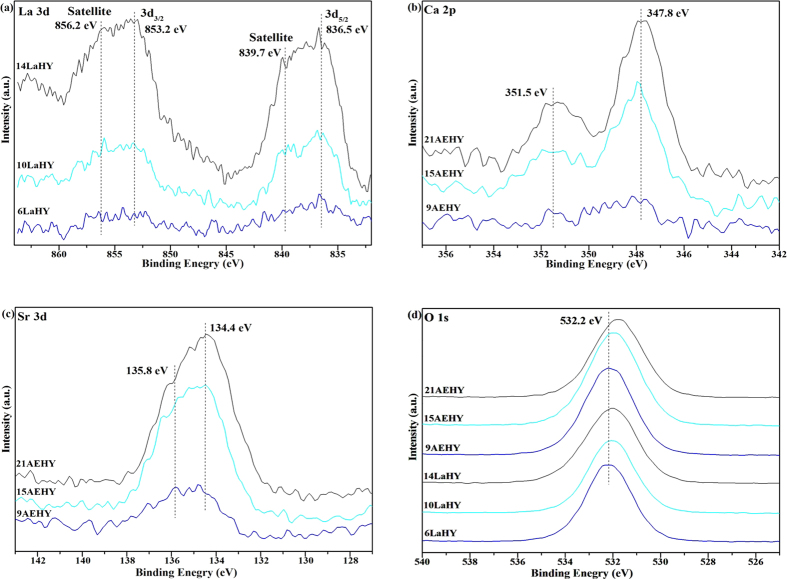
The XPS results of partial samples.

Further investigation for the chemical composition and oxidation states of the prepared samples was obtained by XRF and XPS techniques. The XRF and XPS results are shown in Table 3 and Fig. 4, respectively, for partial samples. (Here, the unit cell formulas of different zeolite samples (without H_2_O) are given in Table 4). It can be seen from Table 3 that the real contents of La are similar with corresponding theoretical contents calculated by unit cell formulas for LaHY zeolites which have been detected by XRF. In contrast, AEHY zeolites are not optimistic, especially for Sr. All these results further reply that the positioning capability of La^3+^ is better than that of AE^2+^, which is in accordance with the XRD and N_2_-physisorption results. The XPS spectrum of La on the surface of partial LaHY zeolites is shown in Fig. 4a. It is observed that the splitting of the main peaks of La 3d_5/2_ and La 3d_3/2_ are at around 836.5 and 853.2 eV, respectively, and the shake-up satellite peaks of La 3d_5/2_ and La 3d_3/2_ are at 839.7 and 856.2 eV, which indicates that La exists as La^3+^ species^28^. The splitting is due to spin orbit interaction and charge transfer from ligand (O 2p) to the metal (La 4f)^29^. It is easy to understand that the intensities of La increase with the increase of exchanged ion content in HY zeolite. For AEHY zeolites, The Ca 2p spectrum (Fig. 4b) presents a doublet structure and partition into two components. Ca 2p_3/2_ and Ca 2p_1/2_ peaks in AEHY zeolites have binding energy of 347.8 and 351.5 eV, respectively^30^. The XPS data for Ca demonstrate that its oxidation state is 2^+^. The Sr 3d spectrum shown in Fig. 4c depicts peaks at binding energy of 134.4 eV (Sr 3d_3/2_) (which proves the existence of Sr^2+ ^31 and 135.8 eV (Sr 3d_5/2_). The O 1s spectrum in Fig. 4d of the samples shows a single peak at 532.2 eV corresponding to the oxide oxygen (O^2−^) in LaHY and AEHY zeolites^22^.

### Acid content and acid sites characterization (NH_3_-TPD and Py-IR)

Temperature programmed desorption is a popular method for the determination of acid amount of solid catalysts as well as acid strength. For Y zeolites, the low-temperature zone (100–250 °C) of the desorption peak is ascribed to weak acid sites, the high-temperature zone (400–600 °C) corresponds to strong acid sites, and the temperature zone between weak acid sites and strong acid sites (250–400 °C) belongs to medium acid sites^22^. The stronger the bonding force between the NH_3_ molecules diffused into the zeolite channels and the acid sites, the higher the desorption energy that is needed; in this case, acid sites are strong.

**Figure 5:**
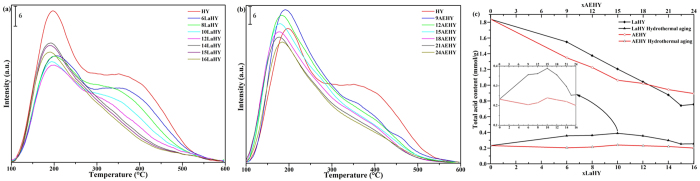
NH_3_-TPD results of (**a**) LaHY, (**b**) AEHY samples and their (**c**) total acid content of fresh and hydrothermal aging.

The NH_3_-TPD results are shown in Fig. 5a,b for LaHY and AEHY samples, respectively, and in 5c for their total acid content of fresh and hydrothermal aging. It can be seen from Fig. 5a that the intensities of strong acid sites gradually decrease as well as weak acid sites and simultaneously the peaks of weak acid sites gradually slightly move to higher temperature with the increase of La^3+^ until 12LaHY, indicating that the entry of La^3+^ can decrease strong acid sites as well as weak acid sites and increase the proportion of medium acid sites. Further increasing the exchanged content of La^3+^, the strong acid sites further decrease (which can be seen from Table 5) while the weak acid sites increase, this may be related to the presence of EFAL, because the presence of EFAL would increases the weak acid content^26^. For AEHY samples shown in Fig. 5b, the intensities of strong acid sites decreased are more severe than that of LaHY zeolite of identical exchanged ion content while the peaks of weak acid sites increase (the increments of weak acid sites gradually decrease with the increase of the exchanged content of AE^2+^) and the peaks of weak acid sites gradually slightly move to lower temperature with the increase of AE^2+^ until 18AEHY. The change of different acid sites is tiny when further increasing the exchanged content of AE^2+^, which is similar with LaHY zeolite. The total acid content of all samples has been given in Fig. 5c. For fresh LaHY zeolites, the total acid content gradually decrease with the increase of La^3+^ and the trend decreased appears linear relationship approximately from 6LaHY to 14LaHY. The acid content decreased of AEHY zeolite is more than that of LaHY zeolite of identical exchanged ion content until 18AEHY while less after 18AEHY. The former indicates that LaHY zeolite reduces the acid content at the same time it is easier to retain a part of acid content compared with AEHY zeolite which may be ascribed to the fact that rare earth cations located in sodalite cage can restrain dealumination and increase a part of acidity through inducing and polarizing H_2_O^16^,^18^; the latter implies that AEHY zeolite can result in the presence of more EFAL than that of LaHY zeolite, whose presence would increases the weak acid content^26^.

**Figure 6:**
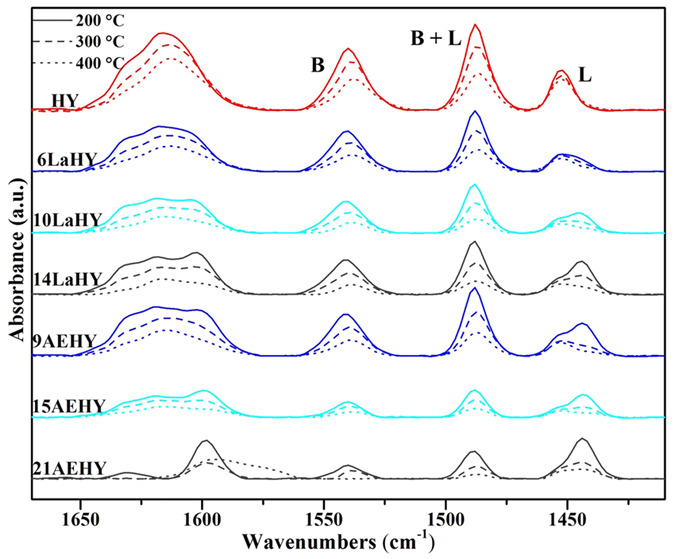
Py-IR spectra of partial samples adsorbed at 200 °C, 300 °C and 400 °C, respectively.

To get further information about the Brönsted and Lewis acid sites, Py-IR spectra results are displayed in Fig. 6 for partial samples and Table 5 for the acidic sites of partial samples determined by Py-IR method. In zeolites, fourcoordinate framework aluminum (FAL) is associated with a Brönsted acid site (SiOHAl), while extra-framework aluminum (EFAL) species generate during the dealumination process act as a Lewis acid site^32^. The wavenumbers of Brönsted and Lewis acid sites are near 1540 and 1450 cm^−1 ^33,34, respectively. As shown in Fig. 6, for HY zeolite, the intensity of Brönsted acid sites (PyB) proportionally decreases while decreases very little for Lewis acid sites (PyL) with the increase of desorption temperature from 200 °C to 400 °C, indicating that PyB consists of weak acid, medium acid and strong acid and PyL is mainly composed of strong acid. The intensity of PyB gradually decrease with the increase of exchanged ions from 6LaHY to 14LaHY and the change of weak acid, medium acid and strong acid with different desorption temperatures is similar with HY zeolite. On the other hand , the intensity of PyL of 6LaHY zeolite dramatically decrease but gradually increase from 6LaHY to 14LaHY. Another change is that the content of strong acid of PyL gradually decrease while the weak acid of PyL gradually increase from 6LaHY to 14LaHY. These results demonstrate that the entry of La^3+^ can modulate strong acid sites as well as weak acid sites according to different exchange content of La^3+^, which is consistent of NH_3_-TPD results. In contrast, the intensity of PyB dramatically decreases while increases very much for PyL especially for weak acid sites with the increase of exchanged ions from 9AEHY to 21AEHY, which implies that the modulation capability of AEHY zeolite to PyB is poor but it can well increase the content of PyL especially for weak acid sites. The quantitative results calculated by empirical formula^34^ are list in Table 5 and the change of values is in good agreement with the above discussions.

According to the literature^35^, the increased acidity of the zeolite due to the reduction of the number of framework Al as well as the presence of EFAL species. Comparing with AEHY zeolite, the PyB and strong acid sites of LaHY zeolite are more, which may be attributed to the partial hydrolysis of hydrated RE ions^36^ according to the following equations:


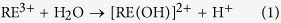



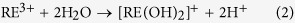


It has been mentioned in NH_3_-TPD results that rare earth cations located in sodalite cage can restrain dealumination and increase a part of acidity through inducing and polarizing H_2_O^16^,^18^. It can be combined with NH_3_-TPD and Py-IR results that the positioning capability in sodalite cage of AEHY zeolite are worse than that of LaHY zeolite and the capability of inducing and polarizing H_2_O of AEHY zeolite in sodalite cage is weak or even almost nonexistent compared with LaHY zeolite. On the other hand, the content of PyL especially for weak acid sites of AEHY zeolite is more than that of LaHY zeolite and the larger the exchanged ion content is, the more obvious the trend shows. Thus, it can be demonstrated that the La^3+^ entered into the sodalite cage can effectively inhibit dealumination in the process of calcination and stabilize the framework of zeolite, whereas, the dealumination takes place due to the poor stability of AE^2+^, whose presence would increases the weak acid content^26^. Similarly, the larger the exchanged ion content is, the more obvious the dealumination takes place and thus the more weak acid sites appear. In terms of the capability of the content of La^3+^ or AE^2+^ in sodalite cage, 12La^3+^ or 18AE^2+^ can be exchanged into one HY zeolite, which can be inferred by the above analyses, namely N_2_-physisorption, XRD, NH_3_-TPD and Py-IR analyses.

### Hydrothermal aging of the samples (XRD and NH_3_-TPD)

**Figure 7:**
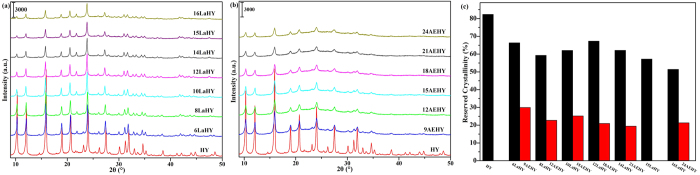
The XRD patterns of (**a**) LaHY, (**b**) AEHY samples and their (**c**) reserved crystallinity of hydrothermal aging.

The hydrothermal aging test had been performed in a tube furnace with 90% water vapor and 10% air at 788 °C for 3 h with the purpose of exploring the thermal and hydrothermal stability of these samples. The results are shown in Fig. 7 for the XRD patterns of (7a) LaHY, (7b) AEHY samples and their (7c) reserved crystallinity of hydrothermal aging and in Fig. 3c for cell parameters of hydrothermal aging. The intensity of the peaks decreases compared with fresh samples (Fig. 3a,b) whatever they are LaHY zeolites or AEHY zeolites of hydrothermal aging, but the extent of decline of peak intensities of AEHY zeolites is much more than that of LaHY zeolites. On the one hand, the results suggest that the occurrence of severe structural damage during the harsh hydrothermal treatment; on the other hand, La^3+^ is effectively helpful in improving the hydrothermal stability of the NaY derived products, which can also be confirmed by the results of reserved crystallinity. The reserved crystallinity of LaHY zeolites is more than twice as much as AEHY zeolites. It is interesting to notice that the reserved crystallinity of HY zeolite reach 82.4%, which is larger than that of all LaHY zeolites. This may be attributed to the rearrangement of HY zeolite resulted from severe dealumination in the process of hydrothermal aging, which can be confirmed by the shrinking result of cell parameters of HY zeolite from 24.53 Å for fresh to 24.28 Å for hydrothermal aging (see Fig. 3c) and the total acid content of HY zeolite of hydrothermal aging (see Fig. 5c). In addition, a fact must be noted that Al-O bond is replaced by Si-O bond and the bond length of the former is longer than that of the latter, thus the cell parameters will shrink.

It can be seen from Fig. 3c that the extent of cell shrinkage gradually decreases from HY to 12LaHY zeolite and it is almost unchanged when further increasing the exchanged content of La^3+^. It is not optimistic for AEHY zeolite, however, the extent of cell shrinkage is much more serious than that of LaHY zeolite, suggesting that the capability of stabilizing the framework of zeolite of AE^2+^ is very poor.

**Figure 8:**
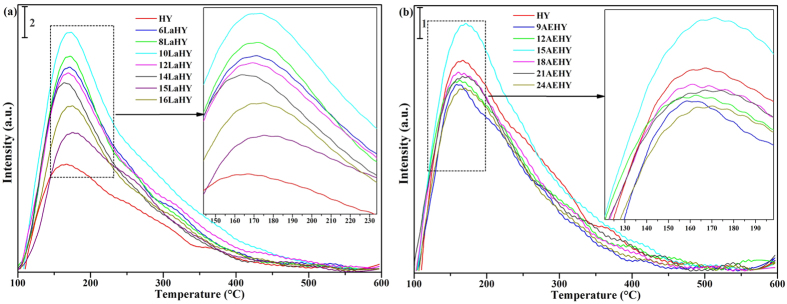
NH_3_-TPD results of (**a**) LaHY, (**b**) AEHY samples of hydrothermal aging.

Figure 8 displays the NH_3_-TPD results of all samples of hydrothermal aging. It can be seen from Fig. 8a that there only exists partial weak acid sites and a small amount of medium acid sites in HY zeolite, indicating the structure and distribution of acid suffer from serious destruction compared with fresh HY zeolite. It is very worth mentioning that the acid sites of LaHY zeolites enhance to some extent and the weak acid sites slightly move to higher temperature. It is the result that the framework of zeolite gets some stability. Among of them, 10LaHY zeolite exhibits the best structure and distribution of acid and exists the best total acid content. After hydrothermal aging, in contrast, for Fig. 8b, the total acid contents of AEHY zeolites are less than that of HY zeolite except 15AEHY zeolite. It is the result that the framework of zeolite gets serious destruction. Thus, it can be demonstrated that La^3+^ is more excellent than AE^2+^ for the relative stability of structure and partial reservation of acid sites, which are the mainly two factors for solid acid catalysis. From the horizontal comparison, no matter what it is La^3+^ or AE^2+^, the acid content reserved is the most when 30 H^+^ are exchanged by La^3+^ or AE^2+^ on one unit cell of HY zeolite. Taking into account the reserved crystallinity treated by hydrothermal aging, 30 H^+^ exchanged on one unit cell of HY zeolite, namely 10LaHY or 15AEHY zeolite, may be optimum exchanged ion content in this study for the modification from NaY to HY zeolite.

### The catalytic cracking of cyclohexane

**Figure 9:**
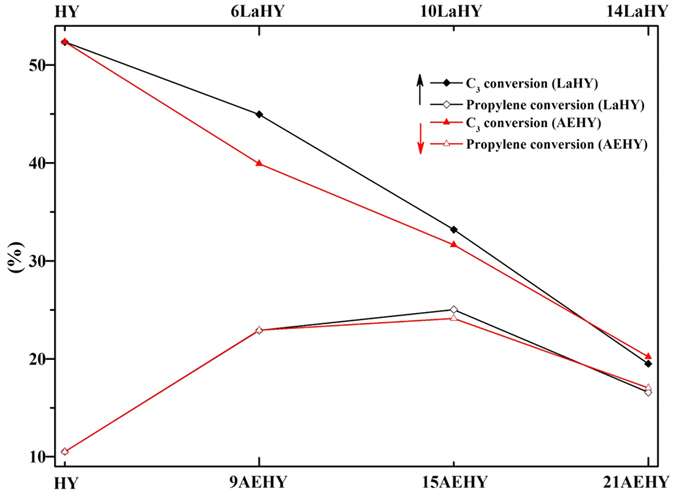
The catalytic cracking of cyclohexane of partial samples.

The catalytic cracking of cyclohexane of partial samples was performed and the results had been displayed in Fig. 9. It can be seen that the conversion of C_3_ (propane and propylene) is the highest for HY zeolite, but the conversion of propylene is the lowest, only about 10%, which suggests that too much acid does not benefit from the formation of propylene. The conversions of C_3_ gradually decrease with the increase of the amount of exchanged ions for both LaHY and AEHY zeolites. The importance is that the conversions of propylene are increasing and reach maximum for both LaHY and AEHY zeolites, demonstrating that appropriate acid amount and strength favor the formation of propylene. In addition, one point must be noted that the conversion of propylene of 10LaHY zeolite is higher than that of 15AEHY zeolite (the exchanged amounts of H^+^ are the same for both 10LaHY and 15AEHY zeolites) and the largest conversion is more than 25%. Similar trend can also be observed in C_3_ conversion. Single on this point of view, La^3+^ is more suitable for the catalytic cracking of cyclohexane than that of AE^2+^.

### The coordination state and stabilization of ion exchanged Y zeolites

**Figure 10:**
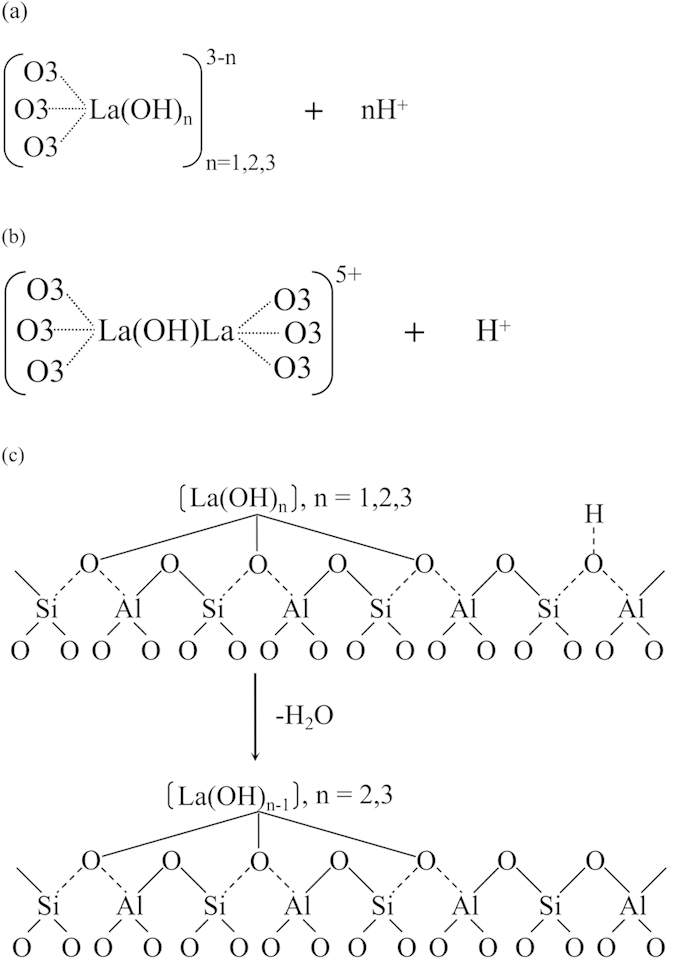
Coordination state and stabilization of LaHY zeolites.

**Figure 11:**
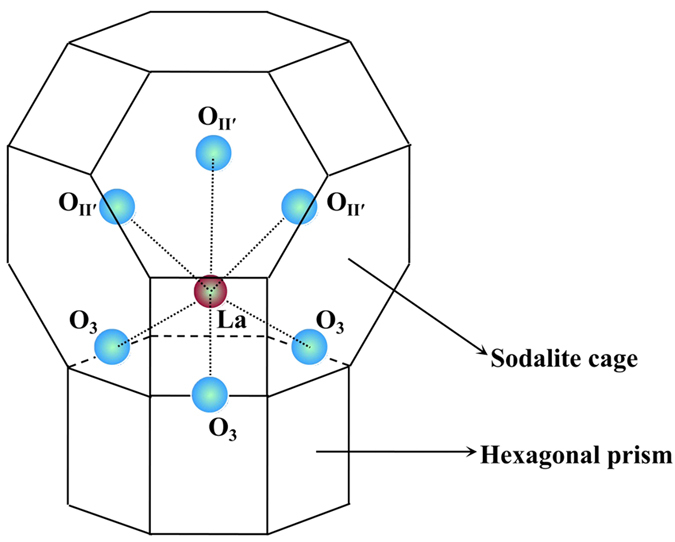
La-O coordination in sodalite cage. (Only one La-O coordination has been given, O3 and O II′ refer to framework oxygen and oxygen on II′ sites, respectively.)

For LaHY zeilites, in the process of La^3+^ exchange, the La^3+^ usually exist as La(H_2_O)_n_^3+^ located in supercages. These ions partially strip off their hydration shells and migrate from cation positions in the supercages to SI’ positions in sodalite cages and coordinate with the framework oxygen (O3). If only one La atom is located in the sodalite cage, the coordination state of the La^3+^ can probably be represented as Fig. 10a. Three H^+^ are replaced by the coordination of one La^3+^ and three O3, and another H^+^ is generated from polarization of water. Under the same degree of dehydration, for LaHY and HY zeolite with the same framework Si/Al ratio, the acid content of LaHY zeolite will be lower and the acid strength will be higher. In theory, therefore, the number of Brönsted acid sites is equal to the number of framework Al atoms; for LaHY zeolites, the number of Brönsted acid sites should be less than the number of framework Al atoms. This inference is consistent with the results reported in the literature^37^. The formation of bridged hydroxyls has been reported for high exchange degrees when one sodalite cage contains two or more RE ions^38^,^39^. In this system, the H^+^ ions from six Al-OH groups were replaced by two La^3+^, producing only one new H^+^ (Fig. 10b), with a high dehydration degree. In this case, the zeolite stability increased. HY zeolites usually have some La^3+^ content, mainly for thermal stability reasons, for adjusting the acidity and stability. This is in reasonable agreement with the related literature^40^. The mechanism of La^3+^ stabilization of Y zeolites is as follows^36^. (i) For Y zeolites containing no La^3+^, structural collapse occurs during hydrothermal treatment as a result of dehydroxylation of sites close to Al. If the Si/Al ratio of zeolites is high, where all the acid sites are relatively isolated, the dehydroxylation reaction is less favored, so high Si/Al ratio zeolites exhibit higher hydrothermal stabilities. (ii) For La-modified HY zeolites, the La^3+^ cations are located in sodalite cages and coordinated to framework oxygen (O3). The H^+^ from the OH-Al are replaced by La^3+^ cations, preventing dehydroxylation of neighboring Al hydroxyls. In contrast, dehydroxylation of LaHY zeolite occurs between the hydroxyls of La species and the neighboring Al-OH, deducing framework vacancies (Fig. 10c). The specific coordination position of La^3+^ in sodalite cage can be seen in Fig. 11.

**Figure 12:**
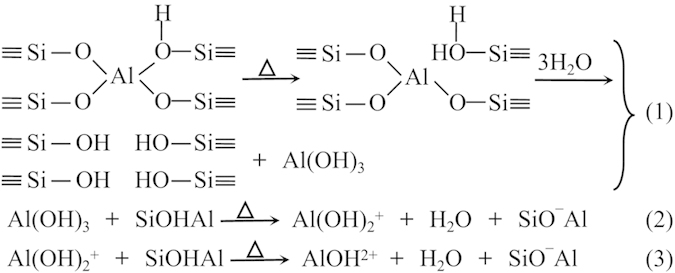
The possible process of dealumination of HY zeolite.

For AEHY zeolites, the capability of AE^2+^ moving to sodalite cage, coordinating with oxygen, and inducing and polarizing H_2_O is poor, thus it is easy for the occurence of dealumination in the process of thermal or hydrothermal. The process of dealumination is similar to that of HY zeolite to great extent and displays in Fig. 12 30. As revealed by previous researches^41^,^42^, during the initial stage of the dealumination of Y zeolite, three-coordinate FAL in the vicinity of an SiOH group is formed due to breaking of framework Si-O-Al bridges (see step (1)), and it can host water molecules, which give rise to octahedrally coordinated Al species. Adsorption of ammonia can convert the coordination of the Al species from octahedral to tetrahedral, which is accompanied by a subsequent healing of the framework Si-OAl bridges. Increasing the degree of dealumination of zeolite Y causes successive hydrolysis of three-coordinate FAL and subsequent formation of extra-framework octahedral Al species, such as Al(OH)_3_ (see step (1))^43^,^44^. Since the fourcoordinate FAL (SiOHAl) is in close proximity to the sixcoordinate EFAL species Al(OH)_3_, it is reasonable to expect that the acidic nature of the former and the basic nature of the latter would lead to easy elimination of a water molecule between them on further increasing the calcination temperature to the higher, for example to 600 °C, with formation of Al(OH)_2_^+^ (see step (2)) or four-coordinate EFAL species AlOH^2+^, which is formed by elimination of one water molecule between SiOHAl and Al(OH)_2_^+^ (see step (3))^32^.

## Conclusions

The metal ions exchange performance of LaHY and AEHY zeolites was studied in this work. HY zeolite with ultra low content Na can be obtained from NaY zeolite through four exchange four calcination method. The positioning capability of La^3+^ in sodalite cage is much better than that of AE^2+^ and about 12 La^3+^ can be well coordinated in sodalite cages of one unit cell of Y zeolite. The acid content including strong acid sites and weak acid sites can be well changed together by LaHY zeolites and PyB and PyL can be gradually decreased and increased, respectively, with the increase of exchanged content of La^3+^. Whereas, the acid content of strong acid sites is dramatically decreased while the weak acid sites is gradually increased and PyB is dramatically decreased while increased very much for PyL especially for weak acid sites with the increase of exchanged content of AE^2+^. Appropriate acid amount and strength favor the formation of propylene and La^3+^ is more suitable for the catalytic cracking of cyclohexane than that of AE^2+^. In terms of hydrothermal stability, LaHY zeolite is much better than AEHY zeolite, the reserved acid content is the most when 30 H^+^ are exchanged by metal ions for one unit cell of HY zeolite whatever it is La^3+^ or AE^2+^, but the acid content of the former is almost twice as much as the latter. In addition, there is one point to be stressed that LaHY zeolites can gradually decrease the acid content of strong as well as weak and AEHY zeolites can sharply decrease strong acid content and can also gradually increase weak acid content. Considering the above factors, we believe that LaHY and AEHY zeolites could act as alternative catalysts for various strong acid catalysis, weak acid catalysis, and even base catalysis due to their unique physicochemical properties.

## Methods

### Samples preparation

The NaY zeolite with a fixed molar ratio of silica to alumina (SiO_2_/Al_2_O_3_ = 5.4) were chosen as the starting material for the preparation of different ion exchanged HY zeolites in this study. A certain amount of NaY zeolite and NH_4_NO_3_ aqueous solution (1 mol/L) with the solid-liquid ratio of 1 : 10 were mixed in a three necked flask and heated to 90 ± 2 °C. The resulting mixtures were kept in stirring for 4 h and then were hot filtrated and multi-washed. The filter cake was broken at 90 °C and then was dried at 140 °C for 4 h. Then the powders were calcined in a muffle stove at 550 °C in flowing air for 4 h. The resulting sample then was repeated the above operations three times, and the final sample was HY zeolite. After the no exchange of sodium content was detected by atomic absorption spectrophotometer^45^, the unit cell formula of HY zeolite was calculated. Here, it is easy to know that the unit cell formula of NaY zeolite is Na_51.9_Si_140.1_Al_51.9_O_384_. After the no exchange of sodium content was detected, the unit cell formula of HY zeolite could be determined and it is H_50.895_Na_1.005_Si_140.1_Al_51.9_O_384_. Similarly, the cell formulas of LaHY and AEHY zeolites would be calculated. Then, 6, 8, 10, 12, 14, 15 and 16 La^3+^ (precursor: La(NO_3_)_3_·6H_2_O) were exchanged with one unit cell of HY zeolite by equal volume impregnation method and the exchanged samples were denoted as 6LaHY, 8LaHY, 10LaHY, 12LaHY, 14LaHY, 15LaHY and 16LaHY, respectively. Take the 6LaHY sample as an example, the mass fraction of 6La^3+^ in one unit cell of HY zeolite could be calculated after the unit cell formula of 6LaHY zeolite was calculated. Then the content of La^3+^ in a certain amount of HY zeolite could be transferred into that in La^3+^ aqueous solution as required by equal volume impregnation. Afterwards, equal volume amount of La^3+^ aqueous solution was impregnated into the HY zeolite drop by drop under stirring and the 6LaHY precursor could be obtained. The other samples were prepared in a similar way. Similarly, 9, 12, 15, 18, 21 and 24 AE^2+^ (AE^2+^ are the mixture of Ca^2+^ and Sr^2+^ (precursors: Ca(NO_3_)_2_·4H_2_O and Sr(NO_3_)_2_), whose molar ratio was 1:1) were exchanged with one unit cell of HY zeolite by equal volume impregnation method and the exchanged samples were denoted as 9AEHY, 12AEHY, 15AEHY, 18AEHY, 21AEHY and 24AEHY, respectively. (explanation: all the exchanged samples were dried at 90 °C for 2 h and then were calcined in a muffle stove at 550 °C in flowing air for 4 h.)

### The test of catalytic cracking

The catalytic cracking of cyclohexane was performed in a micro fixed bed reactor (N_2_ was regarded as carrier gas.). The filling quantity of sample was 0.5 g (20–40 M), reaction temperature was 630 °C. WHSV = 4 h^−1^.

### Hydrothermal aging of the samples

A certain amount of samples were put into a tube furnace with 90% water vapor and 10% air at 788 °C for 3 h. The treated samples were catalysts after hydrothermal aging.

### Sample characterization

X-ray diffraction (XRD) patterns are obtained on a D/MAX-RB X-ray diffractometer (Rigaku, Japan) using Cu Kα (*λ* = 0.15418 nm) radiation. The 2*θ* scans cover the range 10–50° with a scan rate of 1°/min, and the accelerating voltage and applied current are 40 kV and 100 mA, respectively. Textural characteristics of these samples were obtained by nitrogen adsorption at 77 K on a Micrometrics TriStar II 3020 analyzer, using the Brunauer-Emmet-Teller (BET) method for the specific surface area and the Barrett-Joyner-Halenda (BJH) method for the pore distribution. Prior to each analysis, approximate 0.1 g of a catalyst sample was degassed in a N_2_/He mixture at 300 °C for 3 h. X-ray fluorescence spectroscopy (XRF) was made using a ARL-9800 spectrometer. X-ray photoelectron spectroscopy (XPS) measurements were performed in a PHI 5000 VersaProbe spectrophotometer (PE, USA). All binding energies were referenced to the C 1s peak at 284.6 eV of the surface adventitious carbon. NH_3_-TPD was carried out on automated chemisorption analyzer (Finetec Instruments). First, 100 mg of the sample was heated in He (30 mL/min) from room temperature to 600 °C and held for 40 min, subsequently cooled to room temperature in He atmosphere, then heated to 100 °C and held for 20 min, after that, switched to the stream of 5 vol.% NH_3_/He (30 mL/min) and held for 0.5 h under 100 °C. Then, it was purged by He for 0.5 h for removal of residual NH_3_. Then the sample was heated from 100 °C to 600 °C in helium at a heating rate of 10 °C/min. The consumption of NH_3_ was continuously monitored and absorbed with a thermal conductivity detector and hydrochloric acid, respectively. The total acid content of every catalyst was titrated with potentiometric titrator. The IR spectra of adsorbed pyridine were recorded on a Thermo Nicolet Nexus 470 spectrometer equipped with a heatable and evacuatable IR cell containing CaF_2_ windows.

## Additional Information

**How to cite this article**: Deng, C. *et al*. The effect of positioning cations on acidity and stability of the framework structure of Y zeolite. *Sci. Rep.* **6**, 23382; doi: 10.1038/srep23382 (2016).

## Figures and Tables

**Table 1:** Main crystallographic sites in faujasites and correspondent number of atoms.

**S1**	**S2**	**S3**	**S4**	**S5**
–	*20.2 Na*	*32 H*_*2*_*O*	*0.8 La*	*5.0 La*
9.8 Al	10.4 La	32 H_2_O	20.2 Na	–

Adapted from^46^.

Italic: before calcination.

Normal: after calcination.

**Table 2:** Textural properties of the samples.

Samples	BET surface area (m^2^ g^−1^)	Pore volume (cm^3^ g^−1^)	Average pore diameter (nm)
NaY	689.8	0.328	1.90
HY	732.7	0.382	2.08
6LaHY	632.0	0.344	2.18
8LaHY	615.1	0.317	2.06
10LaHY	598.1	0.311	2.08
12LaHY	580.4	0.302	2.08
14LaHY	558.2	0.304	2.18
15LaHY	545.3	0.283	2.18
16LaHY	529.5	0.286	2.17
9AEHY	627.3	0.344	2.19
12AEHY	611.9	0.326	2.13
15AEHY	557.0	0.309	2.22
18AEHY	531.7	0.300	2.23
21AEHY	514.3	0.277	2.15
24AEHY	497.8	0.271	2.18

**Table 3:** Element components detected by XRF.

**Samples**	**XRF (wt.%)**			
	**La**	**Ca**	**Sr**	**Na**
HY	–	–	–	0.14 (0.20)^b^
6LaHY	6.62 (6.74)^a^	–	–	–
10LaHY	10.82 (10.76)^a^	–	–	–
14LaHY	14.07 (14.47)^a^	–	–	–
9AEHY	–	1.70 (1.49)^a^	0.98 (3.25)^a^	–
15AEHY	–	2.72 (2.41)^a^	1.61 (5.26)^a^	–
21AEHY	–	3.69 (3.28)^a^	2.69 (7.16)^a^	–

^a^The data in brackets are corresponding theoretical mass fractions calculated by unit cell formulas (see Table 4).

^b^The data in bracket is the mass fraction of Na measured by atomic absorption spectrophotometer.

**Table 4:** The unit cell formulas of different zeolite samples (without H_2_O).

**Samples**	**The unit cell formula**
NaY	Na_51.9_Si_140.1_Al_51.9_O_384_
HY	H_50.895_Na_1.005_Si_140.1_Al_51.9_O_384_
6LaHY	La_6_H_32.895_Na_1.005_Si_140.1_Al_51.9_O_384_
8LaHY	La_8_H_26.895_Na_1.005_Si_140.1_Al_51.9_O_384_
10LaHY	La_10_H_20.895_Na_1.005_Si_140.1_Al_51.9_O_384_
12LaHY	La_12_H_14.895_Na_1.005_Si_140.1_Al_51.9_O_384_
14LaHY	La_14_H_8.895_Na_1.005_Si_140.1_Al_51.9_O_384_
15LaHY	La_15_H_5.895_Na_1.005_Si_140.1_Al_51.9_O_384_
16LaHY	La_16_H_2.895_Na_1.005_Si_140.1_Al_51.9_O_384_
9AEHY	Ca_4.5_Sr_4.5_H_32.895_Na_1.005_Si_140.1_Al_51.9_O_384_
12AEHY	Ca_6_Sr_6_H_26.895_Na_1.005_Si_140.1_Al_51.9_O_384_
15AEHY	Ca_7.5_Sr_7.5_H_20.895_Na_1.005_Si_140.1_Al_51.9_O_384_
18AEHY	Ca_9_Sr_9_H_14.895_Na_1.005_Si_140.1_Al_51.9_O_384_
21AEHY	Ca_10.5_Sr_10.5_H_8.895_Na_1.005_Si_140.1_Al_51.9_O_384_
24AEHY	Ca_12_Sr_12_H_2.895_Na_1.005_Si_140.1_Al_51.9_O_384_

**Table 5:** The acidic sites of partial samples at different temperatures determined by Py-IR method.

**Samples**	**200 °C (mmol/g)**		**300 °C (mmol/g)**		**400 °C (mmol/g)**	
	**B**	**L**	**B**	**L**	**B**	**L**
HY	0.3376	0.1090	0.2747	0.1035	0.1834	0.0965
6LaHY	0.2851	0.0878	0.2082	0.0764	0.1179	0.0613
10LaHY	0.2383	0.1166	0.1562	0.0793	0.0653	0.0527
14LaHY	0.1999	0.1295	0.1211	0.0870	0.0450	0.0500
9AEHY	0.2324	0.1387	0.1582	0.0679	0.0827	0.0492
15AEHY	0.0971	0.1200	0.0688	0.0673	0.0344	0.0440
21AEHY	0.0739	0.1626	0.0349	0.0810	0.0000	0.0540
